# Early postoperative urinary MCP-1 as a potential biomarker predicting acute rejection in living donor kidney transplantation: a prospective cohort study

**DOI:** 10.1038/s41598-021-98135-0

**Published:** 2021-09-22

**Authors:** Hye Ryoun Jang, Minjung Kim, Sungjun Hong, Kyungho Lee, Mee Yeon Park, Kyeong Eun Yang, Cheol-Jung Lee, Junseok Jeon, Kyo Won Lee, Jung Eun Lee, Jae Berm Park, Kyunga Kim, Ghee Young Kwon, Yoon Goo Kim, Dae Joong Kim, Wooseong Huh

**Affiliations:** 1 Division of Nephrology, Department of Medicine, Samsung Medical Center, Sungkyunkwan University School of Medicine, 81 Irwon-Ro, Gangnam-Gu, Seoul, 06351 Republic of Korea; 2 Department of Digital Health, Samsung Advanced Institute for Health Sciences & Technology, Sungkyunkwan University, Seoul, Republic of Korea; 3 Research Center for Materials Analysis, Korea Basic Science Institute, Daejeon, Republic of Korea; 4 Department of Surgery, Samsung Medical Center, Sungkyunkwan University School of Medicine, Seoul, Republic of Korea; 5 Statistics and Data Center, Samsung Medical Center, Seoul, Republic of Korea; 6 Department of Pathology, Samsung Medical Center, Sungkyunkwan University School of Medicine, Seoul, Republic of Korea

**Keywords:** Predictive markers, Biomarkers, Kidney diseases, Renal replacement therapy

## Abstract

We investigated the clinical relevance of urinary cytokines/chemokines reflecting intrarenal immunologic micromilieu as prognostic markers and the optimal measurement timing after living donor kidney transplantation (LDKT). This prospective cohort study included 77 LDKT patients who were followed for ≥ 5 years. Patients were divided into control (n = 42) or acute rejection (AR, n = 35) group. Early AR was defined as AR occurring within 3 months. Serum and urine cytokines/chemokines were measured serially as follows: intraoperative, 8/24/72 h, 1 week, 3 months, and 1 year after LDKT. Intrarenal total leukocytes, T cells, and B cells were analyzed with immunohistochemistry followed by tissueFAXS. Urinary MCP-1 and fractalkine were also analyzed in a validation cohort. Urinary MCP-1 after one week was higher in the AR group. Urinary MCP-1, fractalkine, TNF-α, RANTES, and IL-6 after one week were significantly higher in the early AR group. Intrarenal total leukocytes and T cells were elevated in the AR group compared with the control group. Urinary fractalkine, MCP-1, and IL-10 showed positive correlation with intrarenal leukocyte infiltration. Post-KT 1 week urinary MCP-1 showed predictive value in the validation cohort. One-week post-KT urinary MCP-1 may be used as a noninvasive diagnostic marker for predicting AR after LDKT.

## Introduction

Kidney transplantation (KT) is the final and definitive treatment option for end-stage kidney disease (ESKD) patients. Early prediction of acute rejection (AR) and overall renal allograft function is crucial for appropriate management considering long-term outcome of KT. Although kidney biopsy is the gold standard to diagnose rejection in KT patients^1–3^, it is not convenient or feasible as a follow-up diagnostic tool because of its invasiveness, especially during the immediate postoperative period after KT. Therefore, non-invasive surrogate markers for early diagnosis and treatment of AR are required.

With the advance of molecular biology, a number of biomarkers have been evaluated for prediction, diagnosis, and risk stratification in KT patients. However, there is no consensus regarding specific types of noninvasive biomarkers and timing of sample acquisition in KT patients^4^. Urinary cytokines and chemokines have been reported as promising biomarkers substituting kidney biopsy because urine was expected to contain inflammatory mediators involved in alloimmune responses leading to AR or interstitial fibrosis/tubular atrophy after KT^5–8^. Since the association of pretransplant serum levels of CXCL9 and AR was reported^9^, urinary CXCL9 at 6 months post-KT was suggested as a promising biomarker in KT patients^10^. If specific urine cytokines/chemokines can be used as surrogate markers substituting kidney biopsy, especially during the early postoperative period, it would be very helpful for both KT patients and physicians.

In this study, we aimed to evaluate the diagnostic and prognostic potential of urinary cytokines/chemokines at different time points after KT and to identify specific cytokines/chemokines reflecting intrarenal immunologic micromilieu as well as the optimal sample acquisition time for early diagnosis of AR.

## Results

### Baseline characteristics

Table 1 shows patient baseline characteristics according to the group. Patients’ age ranged from 18 to 69 years (mean: 42.4 years) and 52 patients were male. Three patients received a second KT. A total of 35 patients experienced AR after KT. There was no difference in age or sex between the control and AR groups. Hypertension was more prevalent in the control group while lupus nephritis and autosomal dominant polycystic kidney disease were more prevalent as the underlying disease of ESKD in the AR group. There were no differences in proportions of induction therapy agents and ABO-incompatible KT. The proportions of patients with ABO incompatibility, PRA positivity, and DSA positivity were comparable between the groups.

**Table 1:** Baseline characteristics, induction therapy for kidney transplantation, and immunologic risk factors of the original cohort.

	Total (n = 77)	Control (n = 42)	AR (n = 35)	P value
Age, mean (SD) (years)	42.4 (12.9)	42.0 (13.7)	44.1 (11.9)	0.292
Male, number (%)	52 (67.5)	26 (61.9)	26 (74.3)	0.248
**Cause of ESKD, number (%)**				0.038
Diabetes mellitus	19 (24.7)	9 (24.7)	10 (27.8)	0.469
Hypertension	5 (6.5)	5 (12.2)	0 (0)	0.059
Glomerulonephritis	29 (36.7)	17 (41.5)	12 (33.3)	0.577
Others	10 (13.0)	2 (4.9)	8 (11.1)	0.037
Unknown	14 (18.2)	9 (21.9)	5 (13.9)	0.418
**Induction therapy, number (%)**				0.250
Steroid only	4 (5.2)	3 (7.1)	1 (2.9)	
Basiliximab and steroid	62 (80.5)	33 (78.6)	29 (82.9)	
ATG and steroid	11 (14.3)	6 (14.3)	5 (14.3)	
ABO-incompatible KT, number (%)	4 (5.2)	1 (2.4)	3 (8.6)	0.325
**PRA, class I (+), number (%)**	15 (19.5)	10 (23.8)	5 (14.2)	> 0.999
% in patients with PRA, class I		41.1 ± 29.24	25.4 ± 21.93	
**PRA, class II (+), number (%)**	22 (28.6)	14 (33.3)	8 (22.9)	> 0.999
% in patients with PRA, class II		24.4 ± 31.67	33.2 ± 37.98	
DSA (+), number (%)	6 (7.8)	3 (7.1)	3 (8.6)	> 0.999

Continuous variables are expressed as mean (standard deviation) and categorical variables are expressed as number (percentage).

Others for cause of ESKD included autosomal dominant polycystic kidney disease, lupus nephritis, obstructive nephropathy, and Alport syndrome.

% in patients with PRA: mean ± standard deviation.

*AR* acute rejection, *ATG* anti-thymocyte globulin, *DSA* donor specific antibodies, *ESKD* end-stage kidney disease, *KT* kidney transplantation, *PRA* panel reactive antibodies, *SD* standard deviation.

### Changes in renal function for 5 years after KT

**Figure 1:**
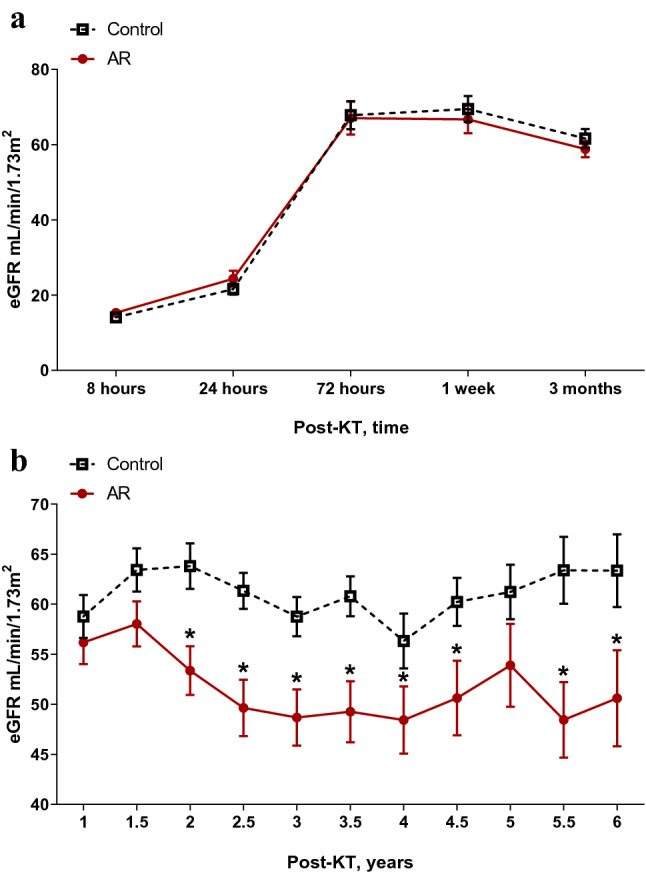
Serial changes in renal function. The eGFR from 2 years after KT was significantly lower in the AR group compared with the control group. *P < 0.05 compared with the control group. *AR* acute rejection, *eGFR* estimated glomerular filtration rate, *KT* kidney transplantation. Data are shown as mean ± standard error of mean.

Changes in renal function were assessed as serial changes in eGFR over 5 years. Renal function of the AR group was comparable with the control group until 3 months after KT. The mean eGFR from 2 years after KT was significantly lower in the AR group compared with the control group. (P < 0.05) (Fig. 1).

### Postoperative serial follow-ups of urinary cytokines/chemokines

**Figure 2:**
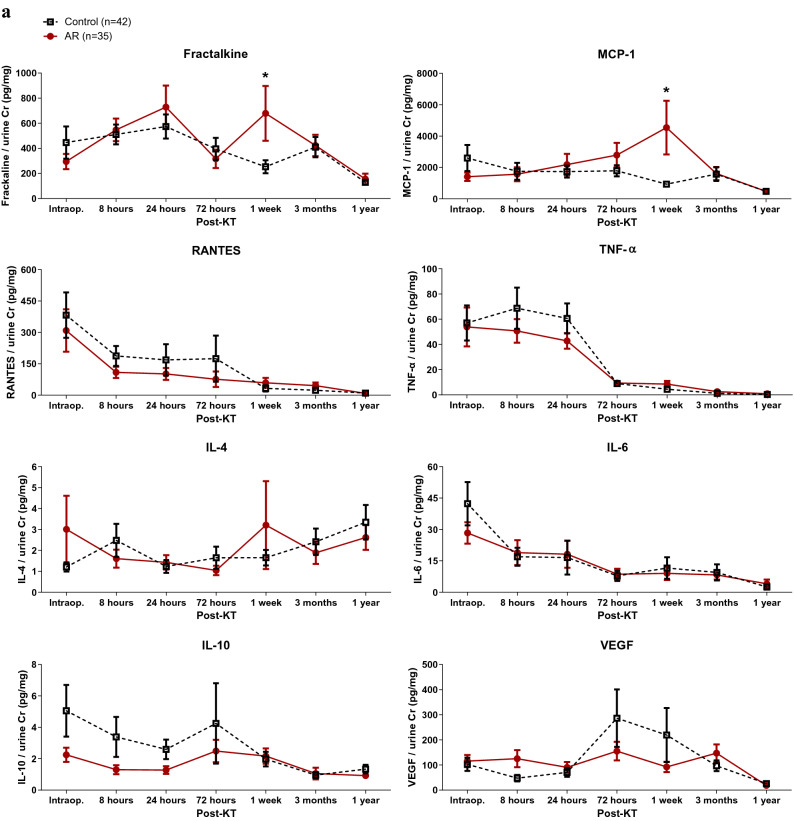

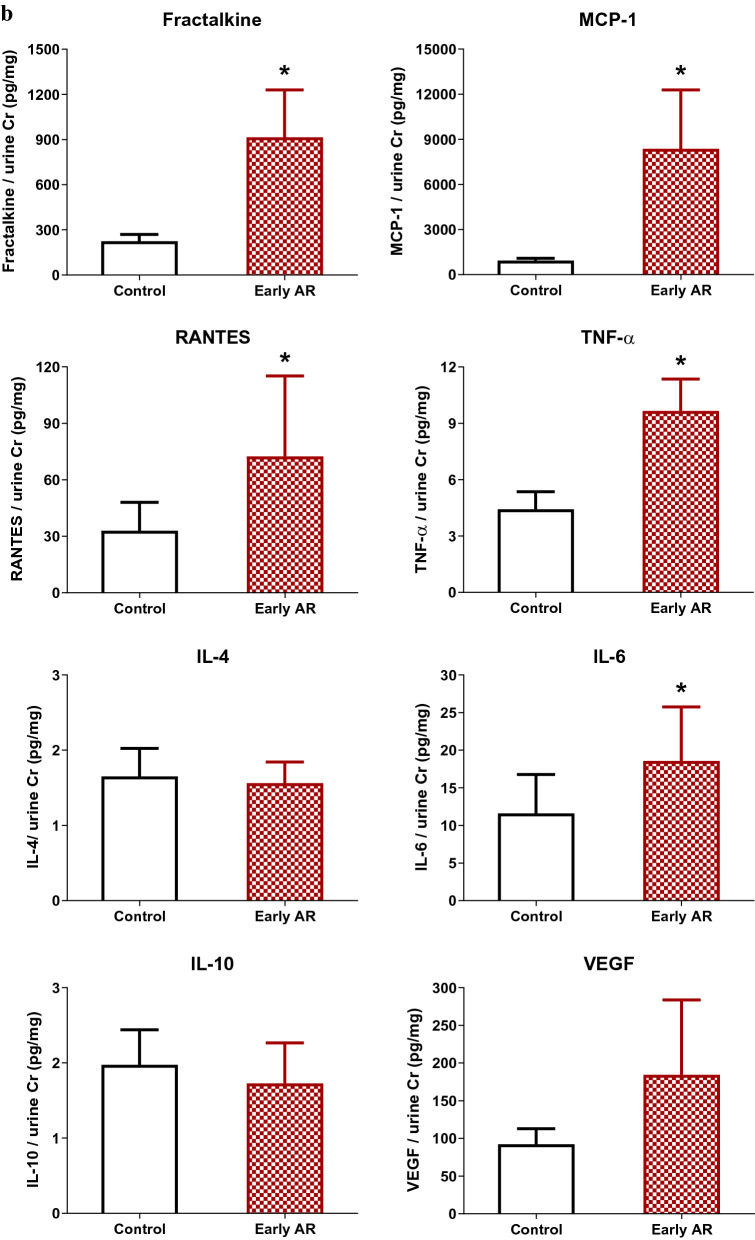
Urinary cytokines/chemokines of the control and AR groups. (**a**) Serial changes in urinary cytokines/chemokines of the control and AR groups. Urinary MCP-1 at 1 week post-KT was significantly higher in the AR group. (**b**) Post-KT 1-week urinary cytokines/chemokines of the control and early AR (AR within 3 months) groups. The early AR group showed higher urinary fractalkine, MCP-1, RANTES, TNF-α, and IL-6 compared with the control group. *P < 0.05 compared with the control group. *AR* acute rejection, *Cr* creatinine, *IL* interleukin, *KT* kidney transplantation, *MCP-1* monocyte chemoattractant protein-1, *RANTES* regulated on activation, normal T cell expressed and secreted, *TNF-α* tumor necrosis factor-α, *VEGF* vascular endothelial growth factor.

Urinary level of MCP-1 at 1 week after KT was higher in the AR group (Fig. 2a). To compare the nearest previous value of urinary cytokines/chemokines from when AR was diagnosed, urinary samples at 1 week after KT (1–9 weeks prior to biopsy) from 13 patients who experienced early AR within 3 months and the control group were compared. Urinary fractalkine, MCP-1, RANTES, TNF-α, and IL-6 at 1 week after KT were significantly higher in patients who were diagnosed with early AR within 3 months compared with the control group (Fig. 2b).

### Postoperative serial follow-ups of serum cytokines/chemokines

**Figure 3:**
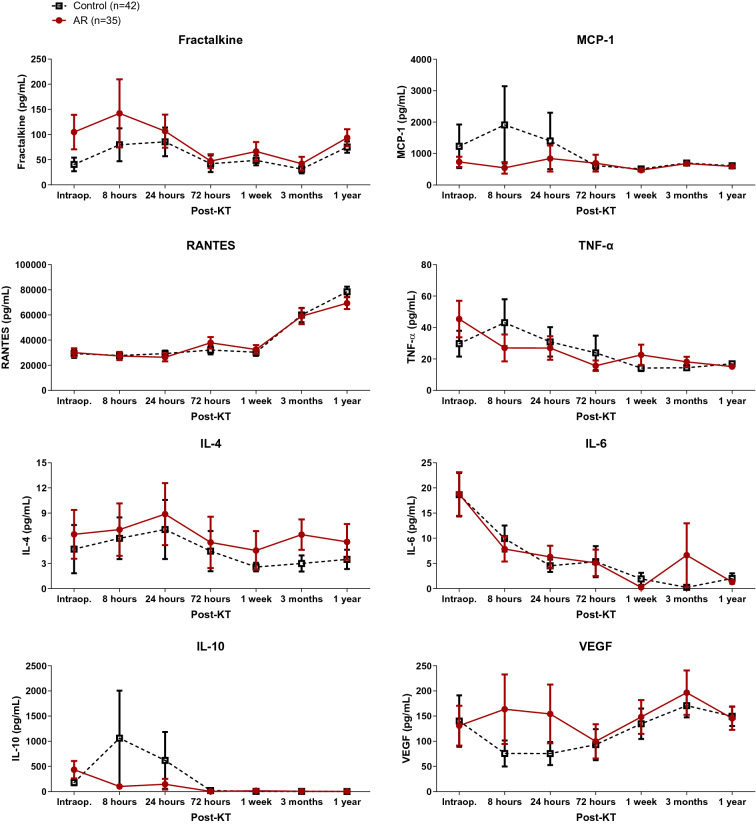
Serial changes in serum cytokines/chemokines of the control and AR groups. Serum cytokines/chemokines were comparable in the control and AR groups at each time point for the first year after KT. *AR* acute rejection, *MCP-1* monocyte chemoattractant protein-1, *IL* interleukin, *KT* kidney transplantation, *RANTES* regulated on activation, normal T cell expressed and secreted, *TNF-α* tumor necrosis factor-α, *VEGF* vascular endothelial growth factor.

Serial changes in serum cytokines/chemokines according to AR are shown in Fig. 3. Serum cytokines/chemokines at all time points were comparable between the control and AR groups. Serum cytokines/chemokines at each time point were not correlated with urinary cytokines/chemokines.

### Correlations between urinary cytokines/chemokines and intrarenal leukocyte infiltration

**Figure 4:**
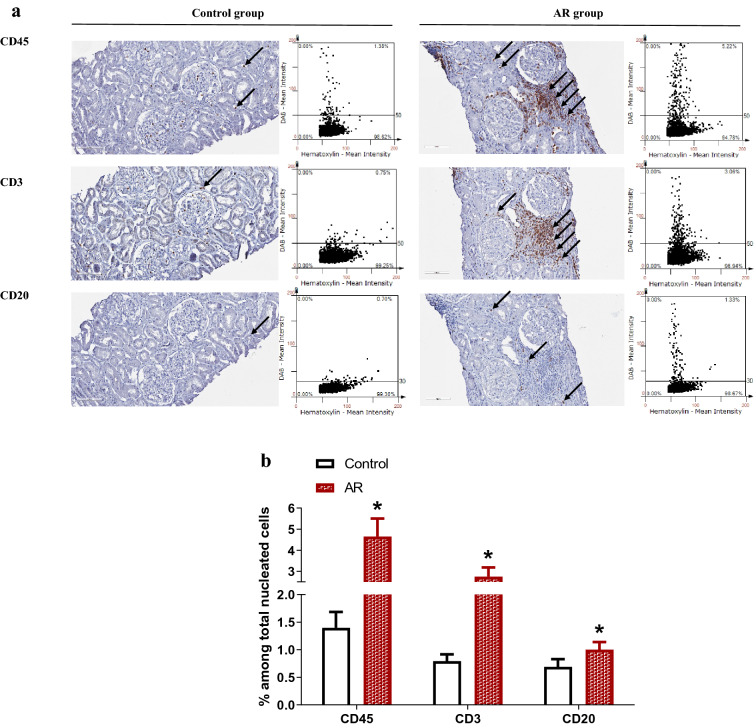
Semiquantitative analysis of intrarenal leukocytes infiltration. (**a**) Intrarenal infiltration of total leukocytes expressing CD45, T cells expressing CD3, and B cells expressing CD20 was compared with immunohistochemistry followed by semiquantitative analysis using tissueFAXS. Arrows indicate CD45-positive leukocytes, CD3-positive T cells, or CD20-positive B cells (×200). (**b**) The AR group displayed a significantly higher infiltration of total leukocytes, T cells, and B cells compared with the control group. *P < 0.05 compared with the control group. *AR* acute rejection, *CD* clusters of differentiation.

Intrarenal infiltration of total leukocytes, T cells, and B cells was analyzed in renal allograft biopsy tissues using immunohistochemistry with anti-CD45, anti-CD3, and anti-CD20. Total leukocytes, T cells, and B cells were significantly elevated in the AR group (Fig. 4).

**Figure 5:**
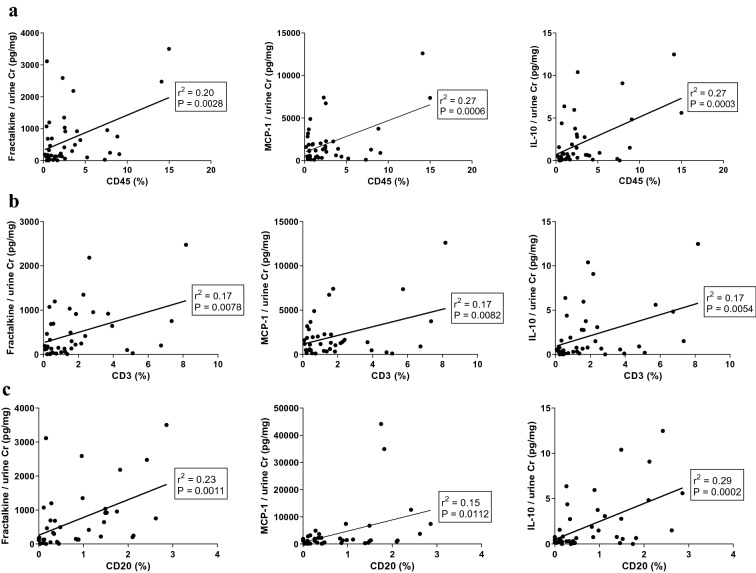
Correlation between intrarenal leukocytes infiltration and urinary cytokines/chemokines. (**a**) Urinary fractalkine, MCP-1, and IL-10 showed positive correlation with infiltration of total leukocytes expressing CD45. (**b**) Urinary fractalkine, MCP-1, and IL-10 showed positive correlation with intrarenal T cells expressing CD3. (**c**) Urinary fractalkine, MCP-1, and IL-10 showed positive correlation with intrarenal B cells expressing CD20. *CD* clusters of differentiation, *Cr* creatinine, *IL* interleukin, *MCP-1* monocyte chemoattractant protein-1.

Urinary fractalkine, MCP-1, and IL-10 showed positive correlation with total leukocyte infiltration. Urinary excretion of fractalkine, MCP-1, and IL-10 was positively correlated with intrarenal T cells and B cells (Fig. 5).

### Potential value of urinary MCP-1 and fractalkine for predicting AR and early AR

Potential predictive value of urinary MCP-1 and fractalkine was further analyzed using sensitivity analysis. The best thresholds for predicting early AR and AR were 1100 pg/mg for urinary MCP-1/creatinine and 300 pg/mg for urinary fractalkine/creatinine. The results of the sensitivity analysis are summarized in Table 2.

**Table 2:** The sensitivity analyses for post-KT 1 week urinary MCP-1 and fractalkine.

	Urinary MCP-1/creatinine		Urinary fractalkine/creatinine	
Threshold (pg/mg)	1100		300	

	AR	Early AR	AR	Early AR
Accuracy (%)	65.7	68.7	62.7	74.6
Sensitivity (%)	61.3	84.6	41.9	61.5
Specificity (%)	69.4	64.8	80.6	77.8
PPV (%)	63.3	36.7	65.0	40.0
NPV (%)	67.6	94.6	61.7	89.4

*AR* acute rejection, *KT* kidney transplantation, *MCP-1* monocyte chemoattractant protein-1, *NPV* negative predictive value, *PPV* positive predictive value.

### Validation of urinary MCP-1 and fractalkine

**Figure 6:**
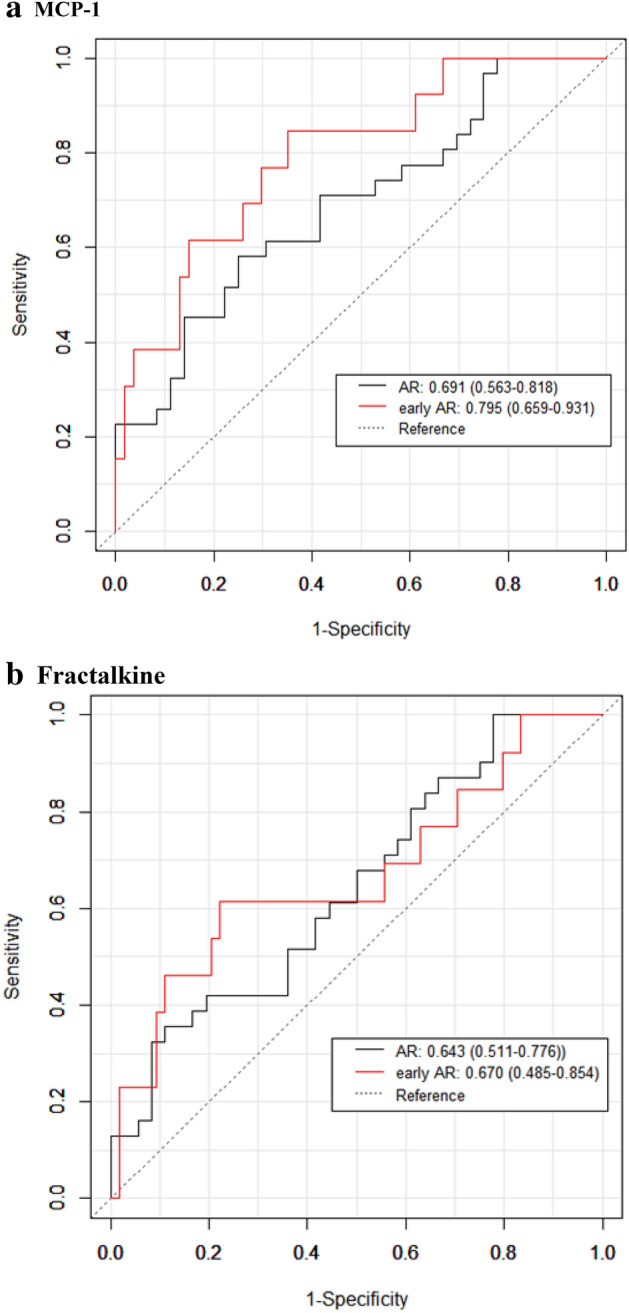
Internal validation of post-KT 1 week urinary MCP-1 and fractalkine. (**a**) AUROC of urinary MCP-1 predicting early AR and AR was 0.795 and 0.691, respectively. (**b**) AUROC of urinary fractalkine predicting early AR and AR was 0.670 and 0.643, respectively. *AR* acute rejection, *AUROC* area under the receiver operating characteristic, *CI* confidence interval, *KT* kidney transplantation, *MCP-1* monocyte chemoattractant protein-1.

Internal validation was performed using bootstrapping (bootstrap B = 1000). The area under the receiver operating characteristic (AUROC) of urinary MCP-1 predicting early AR and AR was 0.795 (95% confidence interval (CI) 0.659–0.931) and 0.691 (95% CI 0.563–0.818), respectively (Fig. 6a). AUROC of urinary fractalkine predicting early AR and AR was 0.670 (95% CI 0.485–0.854) and 0.643 (95% CI 0.511–0.776), respectively (Fig. 6b).

**Figure 7:**
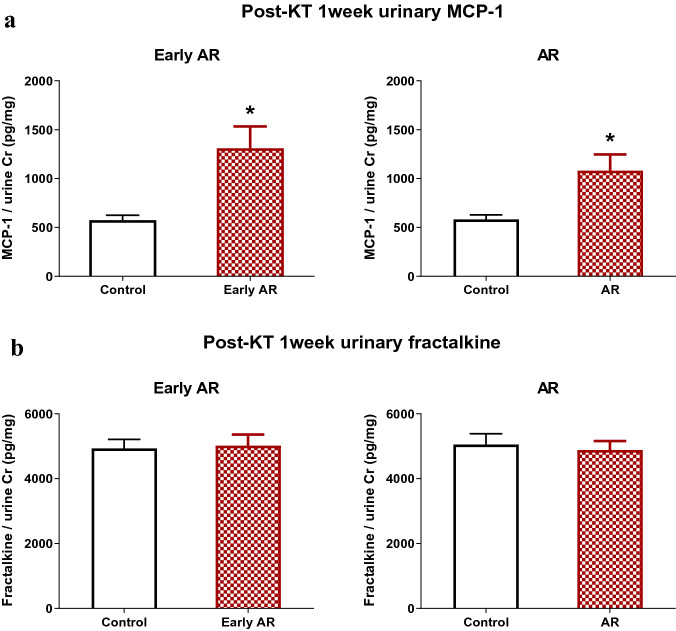
Post-KT 1 week urinary MCP-1 and fractalkine in a validation cohort. (**a**) In the validation cohort including 79 patients, both early AR and AR groups showed significantly higher urinary MCP-1 at 1 week after KT than the control group. (**b**) Post-KT 1 week urinary fractalkine was comparable in both control and AR groups. *AR* acute rejection, *KT* kidney transplantation, *MCP-1* monocyte chemoattractant protein-1.

Urinary MCP-1 and fractalkine were further validated using ELISA in an independent cohort (Table 3). Time separate independent cohort included a total of 79 patients with 38 patients in the control group and 41 patients in the AR group. In the AR group, 28 patients had early AR. A logistic regression of AR and early AR on a continuous, normally distributed variable (X) with a sample size of 79 observations achieves 80% power at a 0.05 significance level to detect an odds ratio of 1.96 and 2.03, respectively, when the prevalence of AR and early AR in the population is 0.52 and 0.35, respectively ^11^. In this independent cohort, post-KT 1 week urinary MCP-1 was significantly higher in the early AR and AR groups than in the control group (Fig. 7a). However, post-KT 1 week urinary fractalkine was comparable in all groups (Fig. 7b).

**Table 3:** Baseline characteristics, induction therapy for kidney transplantation, and immunologic risk factors of the validation cohort.

	Total (n = 79)	Control (n = 38)	AR (n = 41)	P value
Age, mean (SD) (years)	46.9 (11.2)	47.5 (10.7)	46.4 (11.7)	0.655
Male, number (%)	43 (54.4)	18 (47.4)	25 (70.0)	0.263
**Cause of ESKD, number (%)**				0.039
Diabetes mellitus	23 (29.1)	14 (36.8)	9 (22.0)	0.145
Hypertension	7 (8.9)	6 (15.8)	1 (2.4)	0.051
Glomerulonephritis	33 (41.7)	10 (26.4)	23 (56.1)	0.007
Others	7 (8.9)	4 (10.5)	3 (7.3)	0.705
Unknown	9 (11.4)	4 (10.5)	5 (12.2)	1.000
**Induction therapy, number (%)**				> 0.999
Basiliximab and steroid	43 (54.4)	19 (50.0)	24 (58.5)	
ATG and steroid	12 (15.2)	7 (18.4)	5 (12.2)	
Rituximab and steroid	24 (30.4)	12 (31.6)	12 (29.3)	
ABO-incompatible KT, number (%)	15 (20.0)	9 (23.7)	6 (14.6)	0.393
**PRA, class I (+), number (%)**	19 (23.1)	9 (23.7)	10 (24.4)	> 0.999
% in patients with PRA, class I		32.3 ± 29.41	51.3 ± 29.60	
**PRA, class II (+), number (%)**	14 (17.7)	6 (15.8)	8 (19.5)	0.772
% in patients with PRA, class II		29.7 ± 19.55	40.6 ± 31.21	
DSA (+), number (%)	10 (12.7)	3 (7.9)	7 (17.1)	0.3145

Continuous variables are expressed as mean (standard deviation) and categorical variables are expressed as number (percentage).

Others for cause of ESKD included autosomal dominant polycystic kidney disease, lupus nephritis, obstructive nephropathy, and Alport syndrome.

% in patients with PRA: mean ± standard deviation.

*AR* acute rejection, *ATG* anti-thymocyte globulin, *DSA* donor specific antibodies, *ESKD* end-stage kidney disease, *KT* kidney transplantation, *PRA* panel reactive antibodies, *SD* standard deviation.

Reliability of measurements for triplicates of each sample was analyzed with both variability and absolute agreement using coefficient of variation (CV) and intraclass correlation coefficient (ICC), respectively. MCP-1 and fractalkine showed low variability (CV = 0.181 and 0.091), and high agreement (ICC = 0.972 [0.959, 0.981] and 0.956 [0.932, 0.972]) ^12,13^, showing good reliability of measurements for both MCP-1 and fractalkine.

## Discussion

In this study, the diagnostic and prognostic potentials of several serum and urinary cytokines/chemokines were analyzed with serially collected urine samples after LDKT. Urinary MCP-1 at 1 week after KT was identified as an important predictive factor of AR development. Urinary fractalkine, TNF-α, RANTES, and IL-6 at 1 week after KT showed predictive potential for early AR within 3 months post-KT. Urinary fractalkine, MCP-1, and IL-10 exhibited positive correlation with intrarenal total leukocytes, T cells, and B cells. These results suggest clinical usefulness of urinary cytokines/chemokines for early diagnosis of AR in LDKT patients and support the potential role of urinary cytokines/chemokines as surrogate markers of intrarenal immunologic micromilieu.

AR is a serious problem after KT and exerts significant impacts on both allograft and patient survival. Early diagnosis and treatment of AR are crucial for long-term allograft survival. Although kidney biopsy is the gold standard for assessing kidney injury, timely detection of early alterations in allograft immunologic micromilieu has not been feasible with current techniques^14^. Therefore, new biomarkers for predicting changes in intrarenal immunologic micromilieu after KT are required^15^. Ideal biomarkers should be measured reproducibly in noninvasively collected samples. Urine biomarkers are ideal for immune monitoring after KT because they are directly produced by the allograft and can be collected noninvasively and repeatedly. Cytokines and chemokines have been reported to play crucial roles in controlling immune responses within renal allografts^16^. After KT, proinflammatory cytokines are released from allografts and subsequently induce local chemokine secretion^17,18^. Based on urinary cytokines/chemokines can reflect overall changes in intrarenal cytokines/chemokines, our study aimed to find the most appropriate timing and types of urinary cytokines/chemokines during the early postoperative period for predicting AR.

In our study, urinary fractalkine, MCP-1, RANTES, TNF-α, and IL-6 at 1 week after KT were significantly higher in patients who developed early AR within 3 months post-KT. Urinary MCP-1 at 1 week post-KT showed the most prominent increment in early AR patients compared with the control group. Elevation of serum proinflammatory cytokines and chemokines depending on allograft status was previously reported in KT patients^19,20^. MCP-1 and RANTES, important members of the C–C chemokine family, are known to substantially contribute to glomerular and tubulointerstitial damage in diabetic nephropathy through a positive feedback loop of inflammation^21^. Blocking of the MCP-1/CCR2 chemokine pathway was reported to improve survival of islet allografts^22^. Intrarenal tissue expression of RANTES and MCP-1 was elevated in KT patients with chronic allograft dysfunction^23^. Urine MCP-1 was also reported to be elevated in patients with AR^24,25^ or polyomavirus nephropathy^26^. However, most previous studies investigated these cytokines/chemokines using a cross-sectional study design. Our results support these previous findings and further reveal the diagnostic potential of urinary MCP-1 as a surrogate biomarker for predicting AR via serial measurement.

Urinary fractalkine at 1 week after KT showed not only diagnostic potential for predicting early AR, but also a positive correlation with intrarenal T cells and B cells in protocol biopsy. Fractalkine and its interaction with receptors on leukocytes mediate chemoattraction and play an important role in recruiting NK cells into cardiac allografts^27^. Fractalkine expression was significantly higher in the tubular epithelium and endothelium of the allografts in AR patients^28^. Increased excretion of urinary TNF-α and IL-6 was reported in patients with AR^29–31^. Although our findings were consistent with previous reports and further revealed that excretion of these proinflammatory cytokines/chemokines increased early 1 week after KT in patients at risk for AR, external validation of urinary fractalkine using an independent cohort did not show reproducible results. Further studies in a larger cohort may be required to evaluate the clinical value of urinary fractalkine as a biomarker in LDKT patients.

The activity and subsets of intrarenal leukocytes are known to change actively within renal allografts and create a specific intrarenal immune environment such as renal tissue memory T cells^32,33^. Increased infiltrating leukocytes into renal allografts and activated intrarenal T cells were reported in AR^34^. In our study, the AR group showed more enhanced infiltration of total leukocytes, T cells, and B cells into renal allografts. Urinary fractalkine, MCP-1, and IL-10 showed significant correlation with intrarenal infiltration of total leukocytes, T cells, and B cells. A previous study analyzing the mechanisms of AR in a baboon KT model reported a significant correlation between urinary excretion and intrarenal expression of interferon-γ inducible protein-10 and monokine^35^. Our study supports the clinical usefulness of urinary cytokines/chemokines, especially urinary fractalkine and MCP-1, as surrogate biomarkers that reflect the intrarenal immunologic micromilieu.

A few limitations of this study require consideration. First, there were some time differences in the collection of urine samples and kidney biopsies, ranging from 5 days to 1 month, which was inevitable given the prospective study design and serial collection of urine samples. Second, dividing the raw concentration of urinary cytokines/chemokines by urinary creatinine concentration might not be ideal for correcting for differences in urine concentration. Diverse ranges of proteinuria have been reported in KT patients^36–39^, and dividing the raw concentration of urinary cytokines/chemokines by the urinary protein-to-creatinine ratio might be a more appropriate method^40^. However, correction for urinary concentration differences using urinary creatinine concentration has been widely used in previous studies investigating urine biomarkers^41,42^, and using the urinary protein-to-creatinine ratio as a denominator can cause other confounding effects because the total amount of urinary protein excretion per se already includes urinary cytokines/chemokines. We also protocolized the procedures for urine collection, storage, and analysis since standardized management of urine samples is critical for consistent results. Third, some discrepancies in the types of cytokines/chemokines showing correlations with AR or intrarenal leukocytes were found. Further studies including a large cohort of KT patients are required to confirm the types of urinary cytokines/chemokines that more precisely reflect both cellular and humoral factors of intrarenal immunologic micromilieu. Fourth, a power study estimation could not be performed in our original cohort because of the prospective design and serial measurements of multiple serum and urine samples. The validation cohort was designed with a power study estimation to overcome this limitation.

In conclusion, urinary MCP-1 at 1 week after KT can be used as a noninvasive surrogate marker for predicting early AR in LDKT patients. Moreover, positive correlation with intrarenal leukocytes infiltration and urinary fractalkine, MCP-1, and IL-10 suggests the clinical usefulness of these urinary cytokines/chemokines as biomarkers that reflect the intrarenal immunologic micromilieu.

## Methods

### Study design and participants

This prospective cohort study recruited 77 adult patients who underwent living donor KT (LDKT) from April 2011 to August 2013 at Samsung Medical Center and were followed-up for more than 5 years. Adult (≥ 18 years) LDKT patients with no history of cancer whose urine samples were collected at each time point were included in the study. Exclusion criteria were (1) serum samples missing at more than 3 time points, (2) patients with autosomal-dominant polycystic kidney disease who received simultaneous bilateral nephrectomy of native kidneys with LDKT, and (3) significant bleeding or infection during the immediate postoperative period.

Validation cohort for post-KT 1 week urinary MCP-1 and fractalkine included 79 adult patients who underwent LDKT from September 2013 to December 2015. Sample size was determined to achieve 80% power at a 0.05 significance level considering the incidence of AR and early AR ^11^.

This study was approved by the Institutional Review Board of Samsung Medical Center in compliance with the Declaration of Helsinki (IRB number: 2011-06-117). Informed consent was obtained from all participants before enrollment.

### Data collection

Clinical data including baseline characteristics, induction therapy for KT, immunologic risk factors, and serial changes in renal function were collected from the electronic medical record system. Immunologic risk factors included the presence of ABO incompatibility, panel reactive antibodies (PRA), and donor specific antibody (DSA).

Patients who did not experience AR were classified into the control group and those who developed acute cellular rejection were classified as the AR group. Renal allograft biopsy was used as the diagnostic standard and AR was scored according to the Banff criteria^2^. There were no cases of antibody-mediated rejection. Early AR was defined as AR within 3 months. Renal function was compared with the estimated glomerular filtrate rate (eGFR), which was calculated using the Chronic Kidney Disease Epidemiology Collaboration (CKD-EPI) equation.

### Sample collection and cytokine/chemokine assays

Both urine and serum samples were serially collected at the following time points to measure cytokines/chemokines: intraoperative (during KT) and postoperative 8 h, 24 h, 72 h, 1 week, 3 months, and 1 year. The collected urine samples were centrifuged at 2500 rpm for 15 min under − 4 °C. The supernatant of each sample was aliquoted with 1.4 mL of urine and 1.0 mL of serum. After adding 1/100 volume of protease inhibitor and 0.1 M PMSF (Sigma Aldrich, St. Louis, MO, USA) to each sample, all samples were stored at − 70 °C until analysis. A total of 8 cytokines/chemokines were measured in each sample as follows: regulated on activation, normal T cell expressed and secreted (RANTES), fractalkine, interleukin (IL)-4, IL-6, IL-10, monocyte chemoattractant protein (MCP)-1, tumor necrosis factor (TNF)-α, and vascular endothelial growth factor (VEGF). These 8 cytokines/chemokines were measured using a Milliplex MAP human cytokine/chemokine kit (Merck Millipore, Burlington, MA, USA) according to the manufacturer’s instructions. In the validation cohort, urinary MCP-1 and fractalkine were measured using Quantikine ELISA Kits (R&D Systems, Inc. Minneapolis, MN, USA) according to the manufacturer’s instructions. The concentrations of urinary cytokines/chemokines (pg/mL) were normalized by dividing the raw value by urinary creatinine concentration (mg/dL). Therefore, final values of urinary cytokines/chemokines are expressed as pg/mg.

### Quantification of infiltrating immune cells into renal allografts

Renal allograft biopsy was performed between 12 and 14 days after KT as protocol biopsies in 37 patients or when AR was suspected in 9 patients. Immunohistochemical staining with CD45, CD3, and CD20 was performed on formalin-fixed kidney tissues. Sections (4 μm) were deparaffinized with xylene, rehydrated in a graded alcohol series, and then placed in citrate buffer solution (pH 6.0). Slides were placed in a pressure cooker and heated with microwaves for 10 min to enhance antigen retrieval. After cooling, sections were immersed in hydrogen peroxide solution (DAKO, Carpinteria, CA, USA) for 30 min to block endogenous peroxidase and then treated with serum-free protein block (DAKO) at 4 °C overnight. The next day, slides were incubated for 1 h at room temperature with a 1:100 dilution of monoclonal antibody to CD45, CD3, and CD20. After incubation of slides with a mixed solution containing dextran coupled with peroxidase molecules and goat secondary antibody molecules (DAKO) for 30 min at room temperature, 3,3′-diaminobenzidine tetrahydrochloride (DAKO) was applied to the slides to develop a brown color. Then, slides were counterstained with Mayer’s hematoxylin (DAKO). To calculate the percentage of CD45-, CD3-, and CD20-positive cells among total nucleated cells in renal allografts, the whole field of the slide was scanned and analyzed with TissueFAXS. (TissueGnostics, Vienna, Austria)^43–45^.

### Statistical methods

All results were expressed as mean ± standard deviation (SD) or standard error of the mean (SEM) as appropriate. SPSS 24.0 statistical software (SPSS Inc., Chicago, IL, USA) and GraphPad Prism 9 (GraphPad Software, La Jolla, CA, USA) were used for statistical analyses. Group means were compared using the Mann–Whitney test. The Chi-square test or Fisher's exact test was used for comparing baseline characteristics between the control and AR groups. The correlations between each cytokine/chemokine and intrarenal leukocytes, and post-KT 1 week urinary and serum MCP-1 or fractalkine were assessed using the Pearson R correlation. Internal validation of post-KT urinary MCP-1 and fractalkine was performed using bootstrapping (bootstrap B = 1000) by calculating AUROC.

Power analysis was performed using PASS software (PASS 2021: v21.0.2, NCSS Statistical Software, Kaysville, UT, USA)^11^. Reliability of measurements was evaluated with coefficient of variation (CV) and intraclass correlation coefficient (ICC)^12,13^.

Statistical significance was determined when the P value was < 0.05.

## Data Availability

The data that support the findings of this study are available on reasonable requests from the corresponding author.

## Abbreviations

AR: Acute rejection
AUROC: Area under the receiver operating characteristic
CKD: Chronic kidney disease
CI: Confidence interval
CV: Coefficient of variation
DSA: Donor specific antibody
eGFR: Estimated glomerular filtration rate
ESKD: End-stage kidney disease
ICC: Intraclass correlation coefficient
KT: Kidney transplantation
NPV: Negative predictive value
PPV: Positive predictive value
PRA: Panel reactive antibodies
